# The shoulder abductor strength is a novel predictor of tracheostomy in patients with traumatic cervical spinal cord injury

**DOI:** 10.1186/s12891-022-05988-1

**Published:** 2022-11-29

**Authors:** Yunbo Jian, Zhiping Mu, Dawei Sun, Dan Zhang, Chunmei Luo, Zhengfeng Zhang

**Affiliations:** 1 Department of Orthopedics, Xinqiao Hospital, Army Medical University, Shapingba District, 183 Xinqiao Main Street, Chongqing, China; 2 Chongqing Nankai Secondary School, Chongqing, China

**Keywords:** Shoulder abductor strength, Traumatic cervical spinal cord injury, Tracheostomy, Predictor

## Abstract

**Background:**

Early prediction of tracheostomy in traumatic cervical spinal cord injury (TCSCI) patients is often difficult. This study aims to clarify the association between shoulder abductor strength (SAS) and tracheostomy in patients with TCSCI.

**Methods:**

We retrospectively analyzed 513 TCSCI patients who were treated in our hospital. All patients were divided into a tracheostomy group and a non-tracheostomy group. The SAS was assessed using the Medical Research Council (MRC) Scale for Muscle Strength grading. Potential predictors were assessed for their association with tracheostomy in patients. A nomogram was developed based on multivariable logistic regression analysis (MLRA) to visualize the predictive ability of the SAS. Validation of the nomogram was performed to judge whether the nomogram was reliable for visual analysis of the SAS. Receiver operating characteristics curve, specificity, and sensitivity were also performed to assess the predictive ability of the SAS.

**Results:**

The proportion of patients with the SAS grade 0–2 was significantly higher in the tracheostomy group than in the non-tracheostomy group (88.1% vs. 54.8%, *p* = 0.001). The SAS grade 0–2 was identified as a significant predictor of the tracheostomy (OR: 4.505; 95% CI: 2.080–9.758; *p* = 0.001). Points corresponding to both the SAS grade 0–2 and the neurological level of injury at C2-C4 were between 60 and 70 in the nomogram. The area under the curve for the SAS grade 0–2 was 0.692. The sensitivity of SAS grade 0–2 was 0.239. The specificity of SAS grade 0–2 was 0.951.

**Conclusions:**

SAS is a novel predictor of tracheostomy in patients after TCSCI. The SAS grade 0–2 had a good predictive ability of tracheostomy.

## Background

Traumatic cervical spinal cord injury (TCSCI) is a catastrophic injury that can lead to motor and sensory dysfunction, and in severe cases, death [1]. Pulmonary complications are the main cause and even the primary cause of death after TCSCI [2, 3]. So, mechanical ventilation and tracheostomy are required in some patients with TCSCI [4]. Early tracheostomy can increase patient comfort, improve respiratory complications, reduce the number of ventilator days, and shorten Intensive Care Unit (ICU) and hospital stays [5–9]. To take advantage of these benefits and allocate resources accordingly, it is important for surgeons to have a tool to predict whether a patient might need a tracheostomy. However, early prediction of tracheostomy in patients with TCSCI has so far been often difficult [10]. Many scholars have studied the risk factors for tracheostomy after TCSCI, the results showed that forced vital capacity, neurological level of injury (NLI) at C4 or above, MRI scans showing hematoma-like changes, smoking history, advanced age, and an American Spinal Injury Association (ASIA) grade A are all risk factors [11–14]. However, these indicators have some limitations: the clinical application is cumbersome; those who suffer great injury had to receive a tracheostomy may not have acceptable and reproducible pulmonary function test results [15], and there is no standardized bedside predictor.

Previous studies have shown that a grade of ASIA A and an injury level above C4 are the most common predictors of tracheostomy [16, 17]. The shoulder abduction is accomplished by the deltoid in conjunction with the supraspinatus [18]. The deltoid is innervated by the axillary nerve, and the supraspinatus is innervated by the suprascapular nerve. Interestingly, the cervical spinal cord innervating the axillary nerve has some overlap with the cervical spinal cord innervating the phrenic and intercostal nerves, respectively. And the cervical spinal cord innervating the suprascapular nerve also has some overlap with the cervical spinal cord innervating the phrenic and intercostal nerves, respectively. Meanwhile, we believe that the shoulder abductor strength (SAS) is related to the ASIA grade and the NLI to some extent. So, we hypothesize that the SAS may be a novel predictor for tracheostomy after TCSCI. The purpose of this study was to clarify the association between the SAS and the tracheostomy in patients with TCSCI. To the best of our knowledge, this is the first study to investigate the association between the SAS and the tracheostomy in patients with TCSCI.

## Methods

### Patients and data collection

This retrospective study reviewed the electronic medical records of patients with TCSCI who were treated in our hospital from October 2010 to October 2020. All assessments were performed by experienced senior physicians on admission. The assessment of SAS was also performed by experienced senior physicians at the time of admission. The decision to perform a tracheostomy was made by the spine surgeon in conjunction with the ICU physician and was made when prolongation of the mechanical ventilation (MV) was expected, considering the patient's neurologic function, respiratory function, age, concomitant injury, and other factors. Tracheostomy was performed if any of the following criteria were met: (1) the patient was retained in a transoral tracheal tube and failed to evacuate mechanical ventilation after several attempts; (2) the patient had a lot of sputum and poor coughing power, requiring retention of an artificial airway to drain sputum. The inclusion criteria were as follows: (1) clear history of trauma, (2) well-diagnosed cervical spinal cord injury (SCI), and (3) complete medical records. The exclusion criteria were as follows: (1) brachial plexus injury, (2) severe brain injury, (3) multiple traumas, such as rib fractures, hemothorax, and pneumothorax, etc., (4) acute pulmonary trauma, and (5) pulmonary complications (e.g., aspiration pneumonia) and history of lung disease such as chronic obstructive pulmonary disease (COPD). A total of 513 patients were enrolled, of whom 84 underwent tracheostomy.

Data including sex, age, smoking history, ASIA impairment scale grade, NLI, and the SAS were recorded. The SAS was assessed using manual muscle testing, based on the Medical Research Council (MRC) Scale for Muscle Strength grading: total paralysis (grade 0); palpable or visible contraction (grade 1); active movement, full range of motion with gravity eliminated (grade 2); active movement, full range of motion against gravity (grade 3); active movement, full range of motion against moderate resistance (grade 4); and (normal) active movement, full range of motion against full resistance (grade 5) [19]. ASIA impairment scale grade was assessed using the American Spinal Injury Association (ASIA) standards [20]. According to our previous clinical experience, the SAS was divided into two groups: grade 0–2 and grade 3–5. Referring to the previous literature, ASIA grade was divided into two groups: grade A and grade B-D, and NLI was divided into C2-C5 and C6-C8 [21–23].

### Statistical analysis

Pearson’s chi-squared test was used to determine whether there was a difference in recorded categorical variables between the tracheostomy and non-tracheostomy groups. Spearman's rank correlation coefficient was calculated to determine the relationship between tracheostomy and the SAS. MLRA was subsequently performed to identify the predictors which is closely related to the prediction of tracheostomy. The nomogram for tracheostomy was constructed for visualization the predictive ability of the SAS.

Sensitivity and specificity of the SAS were calculated. Receiver operating characteristic (ROC) curves were established to evaluate the performance of the SAS and nomogram. In order to judge whether the visual analysis of SAS by nomogram is reliable, the nomogram was also evaluated: the area under the curve (AUC) and C-index were calculated to test the discrimination of nomogram for tracheostomy. An internal calibration curve was also established to calibrate and assess the predictive ability of the nomogram. We also performed a ROC analysis of ASIA plus NLI together to predict tracheostomy and a ROC analysis of ASIA and NLI plus SAS together to predict tracheostomy. All analyses and nomogram development were performed using R software (version 4.2.0). Statistical significance was set at *p* < 0.05.

## Results

### Demographics

A total of 513 patients were included in this study. The mean time interval between performing a tracheostomy and injury was 6.88 ± 3.18 days. The baseline characteristics of the included patients with TCSCI are shown in Table 1. Of these 513 patients (413 males and 100 females), 179 (34.9%) had a history of smoking. In terms of neurologic status, 174 (33.9%) patients were classified as having ASIA grade A, 116 (22.6%) ASIA grade B, 127 (24.8%) ASIA grade C, and 96 (18.7%) ASIA grade D. The most common NLI was C5 (*n* = 150, 29.2%), followed by C6 (*n* = 115, 22.4%), then C4 (*n* = 112, 21.8%). Among all patients, the SAS grade was as follows: 216 patients (42.1%) had grade 0, 67 (13.1%) grade 1, 26 (5.1%) grade 2, 101 (19.7%) grade 3, 47 (9.2%) grade 4, and 56 (10.9%) grade 5.

**Table 1:** Demographic of all 513 patients with traumatic cervical spinal cord injury

	Count	%
Age		
< 60	375	73.1
≥ 60	138	26.9
Sex		
Female	100	19.5
Male	413	80.5
Smoking history		
Yes	179	34.9
No	334	65.1
Shoulder abductor strength grade		
0	216	42.1
1	67	13.1
2	26	5.1
3	101	19.7
4	47	9.2
5	56	10.9
ASIA scale		
A	174	33.9
B	116	22.6
C	127	24.8
D	96	18.7
Neurological level of injury		
C2	3	0.6
C3	20	3.9
C4	112	21.8
C5	150	29.2
C6	115	22.4
C7	75	14.6
C8	38	7.4

*ASIA* American spinal injury association

In the present study, 84 (16.4%) patients with TCSCI underwent tracheostomy. The comparison of patients who underwent tracheostomy and those who did not is shown in Table 2. The tracheostomy group had a significantly higher proportion of patients that were of advanced age (35.7% vs. 25.2%, *p* = 0.046). At neurological status, there were statistically significant differences in the NLI at C2-C5 (71.4% vs. 52.4%, *p* = 0.001), and ASIA grade A (61.9% vs. 28.4%, *p* = 0.001) between tracheostomy and non-tracheostomy group. The proportion of patients with the SAS grade 0–2 was significantly higher in the tracheostomy group than in non-tracheostomy group (88.1% vs. 54.8%, *p* = 0.001). There were also significantly more patients who have smoking history in the tracheostomy group (*p* = 0.001).

**Table 2:** Comparison of data between patients with and without tracheostomy

	Tracheostomy *n* = 84	Without tracheostomy *n* = 429	*p* Value
Age, n (%)			0.046
< 60	54 (64.3)	321 (74.8)	
≥ 60	30 (35.7)	108 (25.2)	
Sex, n (%)			0.188
Female	12 (14.3)	88 (20.5)	
Male	72 (85.7)	341 (79.5)	
Smoking history, n (%)			0.001
Yes	44 (52.4)	135 (31.5)	
No	40 (47.6)	294 (68.5)	
Shoulder abductor strength grade, n (%)			0.001
0–2	74 (88.1)	235 (54.8)	
3–5	10 (11.9)	194 (45.2)	
ASIA scale, n (%)			0.001
A	52 (61.9)	122 (28.4)	
B-D	32 (38.1)	307 (71.6)	
Neurological level of injury			0.001
C2-C5	60 (71.4)	225 (52.4)	
C6-C8	24 (28.6)	204 (47.6)	

*ASIA* American spinal injury association

### The SAS and tracheostomy rate

Regarding the SAS, 26.4%, 19.4%, and 15.4% of patients with grade 0, grade 1 and grade 2 muscle strength, respectively, underwent tracheostomy. The proportions of patients with the SAS of grades 3, 4, and 5 who underwent tracheostomy were 4.0%, 6.4%, and 5.4%, respectively. The percentage of tracheostomies in patients with TCSCI was correlated with the distribution of the SAS. Overall, the percentage of patients requiring tracheostomy decreased as the SAS grade increased (γ = -0.829, *p* = 0.042) (Table 3).

**Table 3:** Percent of tracheostomy at each shoulder abductor strength grade

	Tracheostomy		Without tracheostomy		Total	
	Count	%	Count	%	Count	%
Shoulder abductor strength grade						
0	57	26.4	159	73.6	216	100
1	13	19.4	54	80.6	67	100
2	4	15.4	22	84.6	26	100
3	4	4.0	97	96.0	101	100
4	3	6.4	44	93.6	47	100
5	3	5.4	53	94.6	56	100
Total	84	16.4	429	83.6	513	100

Gamma = -0.829, *p* = 0.042

### The SAS and other predictors in the nomogram

**Fig. 1:**
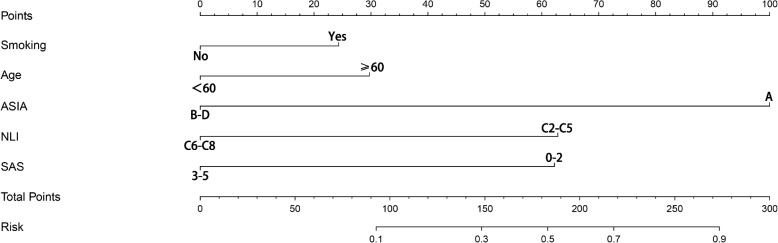
Nomogram to predict the risk of tracheostomy after cervical spinal cord injury. The patient’s score for each risk predictor is plotted on the appropriate scale and vertical lines are drawn from that value to the top points scale to obtain the corresponding scores. All scores are summed to obtain the total points score. The total points score is plotted on the bottom Total Points scale. The corresponding value shows the predicted probability of tracheostomy. C-index = 0.881, SD = 0.039, *P* < 0.05

The results of the MLRA showed that ASIA A (OR = 11.344, *p* = 0.001), NLI at C2-C5 (OR = 4.533, *p* = 0.001), the SAS grade 0–2 (OR = 4.505, *p* = 0.001) and age > 60 (OR = 1.898, *p* = 0.048) were significantly associated with predicting the tracheostomy (Table 4). These predictors and smoking history (OR = 1.798, *p* = 0.051) were included in the nomogram for visual analysis of the effects of the SAS on tracheostomy. In the nomogram, each factor was given a point, and the total number of points was calculated, which corresponded to the risk of tracheostomy. The important finding was that, the points corresponding to the SAS grade 0–2 were between 60 and 70, which suggested that the SAS had pretty good predictive capabilities of tracheostomy. ASIA A carried the most weight, and its corresponding number of points was 100 in the nomogram. The points corresponding to the NLI at C2-C5 were second only to ASIA A, which reflected the NLI had an important impact on the prediction of tracheostomy (Fig. 1).

**Table 4:** Multivariate logistic regression of tracheostomy on the shoulder abductor strength

	β	SE	Wald	*P* value	OR	95%CI
NLI C2-C5	1.511	0.306	24.392	0.001	4.533	2.488–8.257
Age ≥ 60	0.641	0.324	3.913	0.048	1.898	1.006–3.582
Smoking history	0.587	0.301	3.799	0.051	1.798	0.997–3.244
SASG 0–2	1.505	0.394	14.567	0.001	4.505	2.080–9.758
ASIA A	2.429	0.344	49.783	0.001	11.344	5.778–22.272
Constant	-4.396	0.442	98.821	0.001	0.012	

*SASG* Indicates shoulder abductor strength grade, *ASIA* Indicates American spinal injury association, *NLI* Indicates Neurological level of injury

### The evaluation of the SAS

**Fig. 2:**
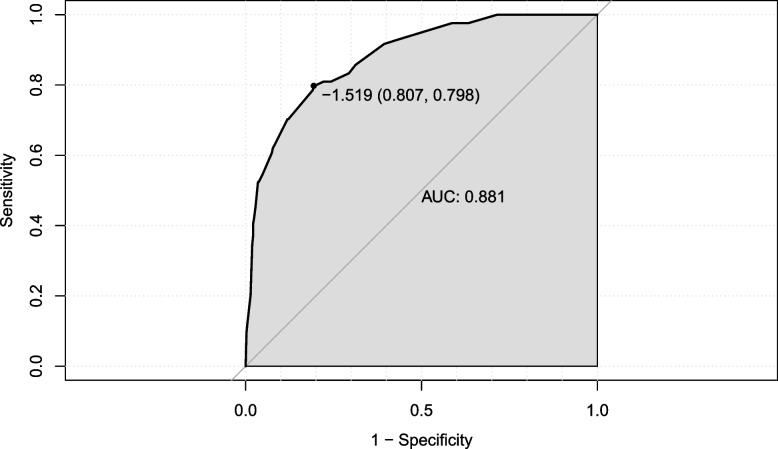
ROC curve for assessing the discrimination of the nomogram in predicting tracheostomy

**Fig. 3:**
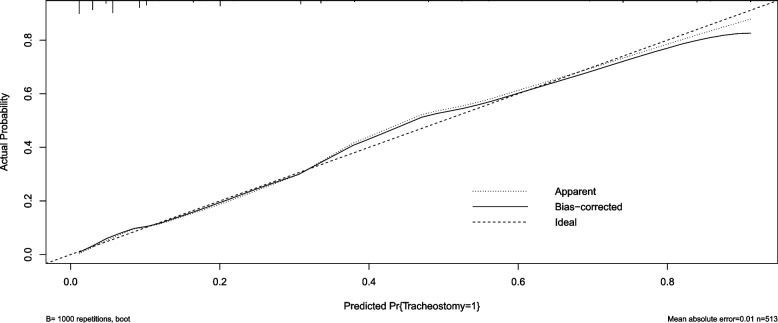
Calibration curve using bootstrap resampling validation (times = 1000) to confirm the prediction performance stability of the nomogram

The nomogram was firstly evaluated. The C-index of the nomogram was 0.881 (SD = 0.039, *p* < 0.05). The ROC curve used to evaluate the performance of the nomogram is shown in Fig. 2. The AUC value was 0.881 (sensitivity = 0.807, specificity = 0.798). The internal calibration curve is shown in Fig. 3. The calibration curves revealed satisfactory consistency, indicating that the nomogram had excellent calibration capabilities. Therefore, the visual analysis of the predictive ability of the SAS for tracheostomy by nomogram was reliable.

**Fig. 4:**
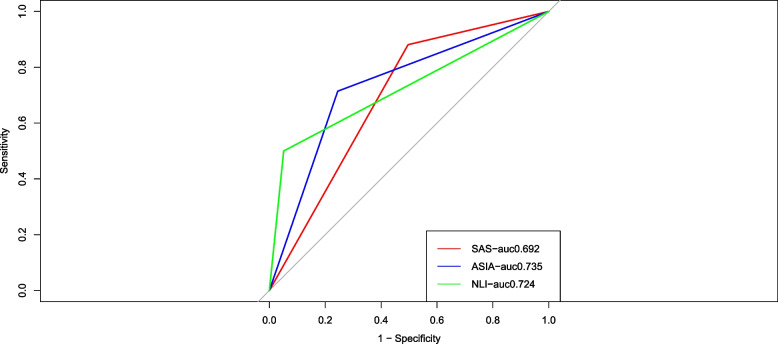
Receiver operating characteristic curve analysis of the SAS and other factors in predicting tracheostomy in patients with TCSCI

**Fig. 5:**
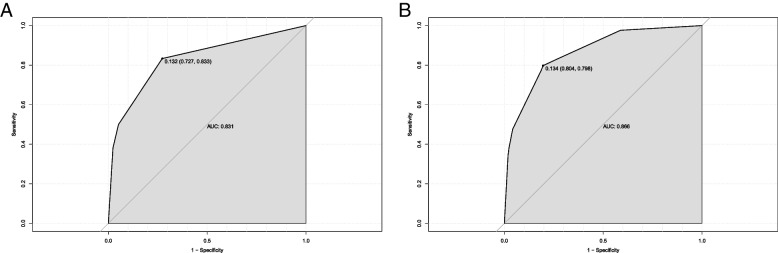
Receiver operating characteristic curve analysis of ASIA grade A plus NLI at C2-C5 together to predict tracheostomy in TCSCI patients (**A**) and receiver operating characteristic curve analysis of ASIA grade A and NLI at C2-C5 plus SAS together to predict tracheostomy in TCSCI patients (**B**)

Further, to examines the predictive value of the SAS for the tracheostomy, the ROC curve analysis was performed. As shown in Fig. 4, the area under the curve for the SAS grade 0–2 was 0.692. The sensitivity of the SAS grade 0–2 was 0.239. The specificity of the SAS grade 0–2 was 0.951. These findings suggest that the SAS grade 0–2 could predict tracheostomy in patients with TCSCI, providing valuable information for the physicians to make treatment decisions. The comparison of the SAS with ASIA and NLI was also performed. The area under the curve for ASIA A was 0.735. The sensitivity of ASIA A was 0.299. The specificity of ASIA A was 0.906. The area under the curve for NLI at C2-C5 was 0.724. The sensitivity of NLI at C2-C5 was 0.211. The specificity of NLI at C2-C5 was 0.895. In Fig. 5A, using only ASIA grade A and NLI at C2-C5 to predict tracheostomy, the AUC was 0.831. In Fig. 5B, using ASIA grade A and NLI at C2-C5 plus SAS grade 0–2 together to predict tracheostomy, the AUC was 0.866.

## Discussion

The SAS grade can be easily assessed using manual muscle test without complicated auxiliary examinations. The manual muscle test has previously been proven to be a reliable and valid examination tool for clinical applications [24]. Consistent with our approach, Sho Ishiwata also used the manual muscle test in one study to measure the SAS [18]. The nomogram clearly showed that a SAS grade 0–2 had a strong predictive ability: the weight was high, and the corresponding points were between 60 and 70. This demonstrates that the SAS is a simple, practical, and reliable predictor of tracheostomy in patients with TCSCI.

The reason of the SAS as a predictor is that, we believe, it combines the characteristics of ASIA Impairment Scale grade and NLI. Respiratory physiology indicates that the central nervous system regulates breathing through the phrenic nerve that controls the diaphragm (the most important respiratory dynamic muscle), and the intercostal nerve controls the intercostal muscle (the secondary auxiliary respiratory dynamic muscle). The shoulder abduction is performed by the deltoid and supraspinatus. The deltoid muscle is completely innervated by the axillary nerve, which arises from segments C4-C6 [25]. The supraspinatus is innervated by the suprascapular nerve, which arises from C5-C6 [26]. Banneheka found that the origin of the phrenic nerve is from segments C3-C5 [27]. The cervical spinal cord segments innervating the intercostal nerves are C4-C5 [28]. The cervical spinal cord innervating the axillary nerve has some overlap with the cervical spinal cord innervating the phrenic and intercostal nerves, respectively. And the cervical spinal cord innervating the suprascapular nerve also has some overlap with the cervical spinal cord innervating the phrenic and intercostal nerves, respectively. Therefore, we speculated that in some emergency situations, the SAS may be used to indirectly and roughly assess the function of the diaphragm and intercostal muscles. This provides an anatomical basis of SAS as a predictor of tracheostomy.

The SAS as a predictor of tracheostomy has several advantages. One advantage is that its predictive ability is considerable, Third to AIS A and NLI at C2-C5. Another advantage is that its clinical application is very simple. Additionally, the SAS can not only be anatomically connected with the phrenic nerve and intercostal nerve, but can also express the severity of cervical SCI to some extent. Therefore, in case of an emergency, the SAS could be used to predict tracheostomy in patients with TCSCI as a simple, practical, and reliable tool.

The ASIA Impairment Scale grade and the level of C5 injury have been shown the most two important risk factors for tracheostomy in patients with TCSCI [11, 13, 14, 29–31]. The present study was consistent with these researches, The OR of ASIA A, the NLI at C2-C5 and the SAS grade 0–2 were 11.344, 4.544 and 4.505, respectively. The AUC of ASIA A, the NLI at C2-C5 and the SAS grade 0–2 were 0.735, 0.724 and 0.692, respectively. In the nomogram, both the SAS grade 0–2 and NLI at C2-C5 corresponded to a score of 60–70, while ASIA grade A corresponded to a score of 100. Compared with the ASIA grade A and the NLI at C2-C5, it demonstrated that the SAS is less predictive of tracheostomy than these two classical factors. However, in some cases, the shoulder abductor strength is also a simple and practical predictor.

Although SAS grade 0–2 is a weaker predictor of tracheostomy than ASIA grade A and NLI at C2-C5, SAS is not intended to replace ASIA or NLI, but rather SAS serves as a new predictor of tracheostomy that complements ASIA, NLI, and other predictors in predicting tracheostomy in TCSCI patients, and together with other predictors predict tracheostomy, allowing for more accurate clinical prediction. This purpose can also be illustrated in the results of this study Fig. 5A and B. In Fig. 5A, using only ASIA grade A and NLI at C2-C5 to predict tracheostomy, an AUC of 0.831 was obtained for the ROC analysis. In contrast, in Fig. 5B, the AUC of the ROC analysis obtained using ASIA grade A and NLI at C2-C5 plus SAS grade 0–2 together to predict tracheostomy was 0.866. This demonstrated that SAS increased the AUC for predicting tracheostomy and that SAS can be clinically helpful in predicting tracheostomy in patients with TCSCI. To make a clinical prediction of tracheostomy more accurate, the more predictors the better. Therefore, this is of great significance that we demonstrated that SAS, a simple and reliable bedside indicator, is a new predictor of tracheostomy.

There are three main limitations to this study. First, this is a retrospective study with a limited level of evidence. Second, the relationship between the shoulder abduction and the respiratory muscles needs further neurophysiological experiments to determine. Third, this study reflected only the experience of a single-specialty spine injury center. Future prospective studies are needed to verify the relationship between the SAS and tracheostomy in patients after TCSCI.

## Conclusion

The SAS was shown to be a novel predictor of tracheostomy in patients after TCSCI. The SAS grade 0–2 had a good predictive ability of tracheostomy. This simple predictor can assist clinicians in making decisions about tracheostomy at the bedside.

## Data Availability

There is identifiable personal information in the data, so the data generated and analyzed during the study are not publicly available. But data are available from the authors upon reasonable request. If anyone would like to request the data from this study, please contact the corresponding author of this study, Zhengfeng Zhang, M.D., Ph.D. (e-mail address: zhangz3@126.com).
